# Cardiac Metastasis Presenting as ST-Elevation Myocardial Infarction

**DOI:** 10.7759/cureus.48183

**Published:** 2023-11-02

**Authors:** Michael J Lewis

**Affiliations:** 1 Internal Medicine, Mercyhealth, Rockford, USA

**Keywords:** cardiac magnetic resonance imaging, st-elevation myocardial infarction, mimic, cardiac tumor, metastatic cancer

## Abstract

A 52-year-old woman with a history of lung cancer presented with progressive shortness of breath. Her ECG showed evidence of ST-elevation myocardial infarction (STEMI) though no evidence of obstructive coronary artery disease (CAD) was seen on coronary angiography. Further imaging with CT and cardiac MRI (CMRI) demonstrated tumor, likely metastatic cancer, within myocardial tissue. This case is demonstrative of the possible relationship between ST-segment elevation on ECG and corresponding tumor invasion and highlights the differential diagnoses of STEMI, including cardiac metastasis.

## Introduction

This is a study of an interesting case of likely cardiac metastasis of primary lung cancer which presented as ST-elevation myocardial infarction (STEMI). Cardiac tumors are typically representative of metastatic cancer, most often the result of a primary lung cancer, and are believed to spread to the heart via lymphatics, a hematogenous pathway, or direct invasion [1]. These tumors are commonly asymptomatic, though some complications may include congestive heart failure, arrhythmia, tamponade, or coronary artery compression [1,2]. Various ECG abnormalities have been reported with these tumors including atrial fibrillation, bradycardia, and/or premature atrial or ventricular contractions [1]. 

This article was previously presented as a poster at the 2023 American College of Cardiology annual meeting on March 5, 2023.

## Case presentation

A 52-year-old woman with a history significant for small cell lung cancer presented to the emergency department (ED) with progressive shortness of breath over the preceding three months which worsened over the five days prior to admission. During the 36 hours prior to her presentation to the ED, the patient also developed substernal chest pressure with radiation to the left side of her chest. The discomfort was worse with exertion as well as laying on her back. The patient reported intermittent nausea, mild diaphoresis, and mild discomfort with palpation of the chest. She noticed that she would become short of breath while walking short distances, and that despite using her oxygen at night, her saturation would drop to 86% at times. She presented normotensive, tachycardic up to 110, afebrile, and with an oxygen saturation of 95% on two liters per minute of nasal canula oxygen.

Approximately one year prior to presentation, the patient had a CT of her chest performed for shortness of breath, which demonstrated a right hilar mass measuring up to 11 centimeters with mediastinal lymphadenopathy. A diagnosis of stage IIIB (T4N2M0) small cell lung carcinoma was eventually made and this was initially treated with a chemotherapy regimen consisting of carboplatin, etoposide, and atezolizumab as well as subsequent radiation therapy. The patient had completed this regimen six months prior to her ED presentation, and, upon subsequent evaluation with her oncologist, her disease had shown stability without progression. Her other medical history included osteoarthritis, anxiety, and a former smoking history of 60-pack-years.

**Figure 1:**
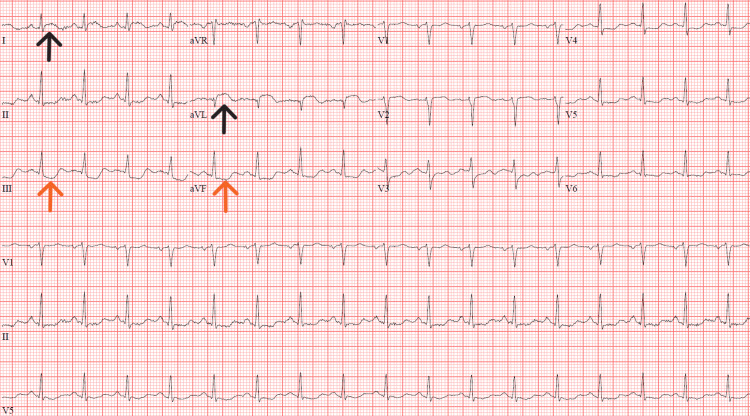
ECG on presentation. ST elevations (black arrows) present in leads I and aVL. ST depressions (orange arrows) noted in leads III and aVF.

ECG on presentation demonstrated ST elevations in leads I and aVL with ST depressions in leads III and aVF (Figure 1). Troponin T was elevated at 102 ng/L (reference range: ≤14 ng/L) and NT-proBNP was elevated at 535 pg/mL (reference range: ≤125 pg/mL). Acute phase reactants were also elevated with sedimentation rate 86 mm/Hr (reference range: 0-30 mm/Hr) and C-reactive protein 12.52 mg/L (reference range: <5.00 mg/L).

**Figure 2:**
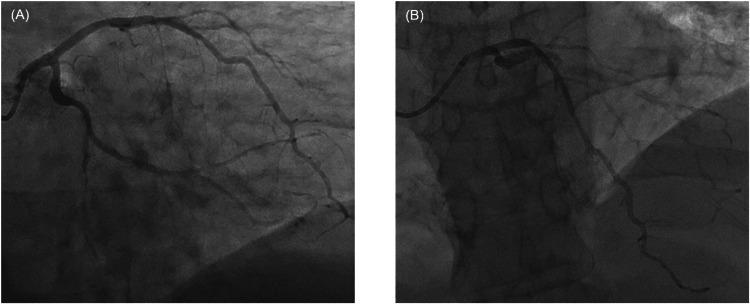
Coronary angiogram images with two coronary angiogram views demonstrating non-obstructive CAD CAD: Coronary artery disease

**Figure 3:**
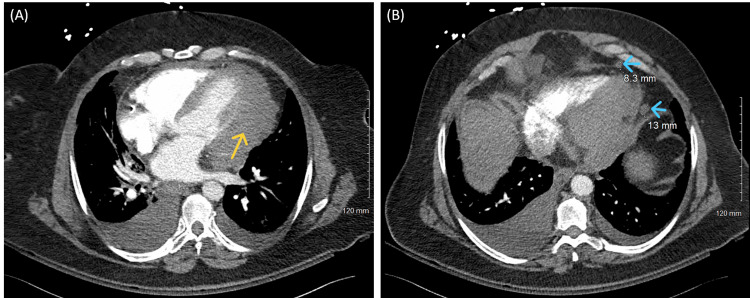
CT angiogram images demonstrating mild lobulated soft tissue density (yellow arrow) contiguous with the left ventricular myocardial wall extending to the left anterior mediastinum (A). Also seen are prominent pericardiac lymph nodes (blue arrows) measuring 8.3 mm and 13 mm (B).

Her symptoms improved slightly with the administration of aspirin 324 mg, 0.5-inch nitroglycerin 2% ointment, morphine 2 mg IV, and metoprolol 5 mg IV. Patient was taken for emergent coronary angiogram which demonstrated mild luminal irregularities of the major coronary arteries, with overall non-obstructive coronary artery disease (CAD) (Figure 2). Given the severity of the patient’s symptoms, CT pulmonary angiography was performed, demonstrating no evidence of pulmonary embolism but rather a soft tissue density of the left ventricle wall with extension into left anterior mediastinum and pericardial adenopathy (Figure 3). A transthoracic echocardiogram performed later during admission did show left ventricular ejection fraction (LVEF) 50-55% with probable moderate hypokinesis of mid-apical and anterolateral segments of the heart with mild concentric left ventricular hypertrophy. There was no evidence of pericardial effusion.

After discharge from the hospital, the patient was asked to continue following up with cardiothoracic surgery for the possibility of excisional biopsy for tumor identification. Repeat ECGs demonstrated no resolution of the abnormal findings noted. Further evaluation with outpatient cardiac MRI (CMRI) demonstrated thickening and invasion of anterior and lateral walls of the left ventricle and adjacent left atrium and right ventricular outflow tract by tumor, felt to represent metastatic tumor related to her small cell lung carcinoma. Unfortunately, this patient's symptoms returned and she was again hospitalized for worsening shortness of breath. Given her poor overall prognosis, the decision was made to pursue hospice care and comfort measures, with the patient passing shortly thereafter.

## Discussion

The differential diagnosis of ST elevation includes those conditions in which the ECG findings would suggest transmural ischemia in the appropriate clinical context. These include acute myocardial infarction, pericarditis, myocarditis, coronary artery spasm, hyperkalemia, pulmonary embolism, Takotsubo cardiomyopathy, early repolarization, Brugada syndrome, cardiac compression, and circumstances with repolarization abnormalities including left bundle branch block, left ventricular hypertrophy, and ventricular pacing [3,4]. Additionally, there have been cases of metastatic cancer, typically of the lung, which appears to mimic or induce cardiac conduction abnormalities including STEMI [5-7]. Cardiac metastases have also been reported to originate from primary leukemia, lymphoma, bronchogenic carcinoma, breast cancer, and esophageal cancer [1].

Tumor cells may spread from other sites to the heart via several different routes including lymphatic or hematogenous spread as well as direct invasion from nearby sites. Metastases may be classified based on invasion location as apical, anteroseptal, anterolateral, or posterolateral with invasion location reflecting upon the ECG with localizing ST-elevation [1]. Aside from ST elevation, cardiac metastasis may result in ST segment depression, low QRS voltage, and T wave flattening or inversion [4]. 

The reasoning for ST elevation in cardiac metastasis has not fully been identified, and not every instance of cardiac metastasis will present with STEMI. More often, patients with cardiac metastasis may be unaware that the cardiac tumor exists, though autopsy examination has demonstrated a larger number of cardiac metastases may exist than documented despite lack of clinical manifestations [7]. This may be due to smaller tumor size without compressive symptoms or the extent of primary tumor metastatic burden and severity of the primary disease compounded with chronic conditions. When ST elevation is present, it may be the result of direct compression of coronary arteries, arterial invasion, electronically inactive tissue invasion, or pericardial infiltration [4,6]. It is postulated that persistent ST elevation without the eventual development of abnormal Q waves may be the result of transmural myocardial damage due to infiltration of tumor cells [1].

**Figure 4:**
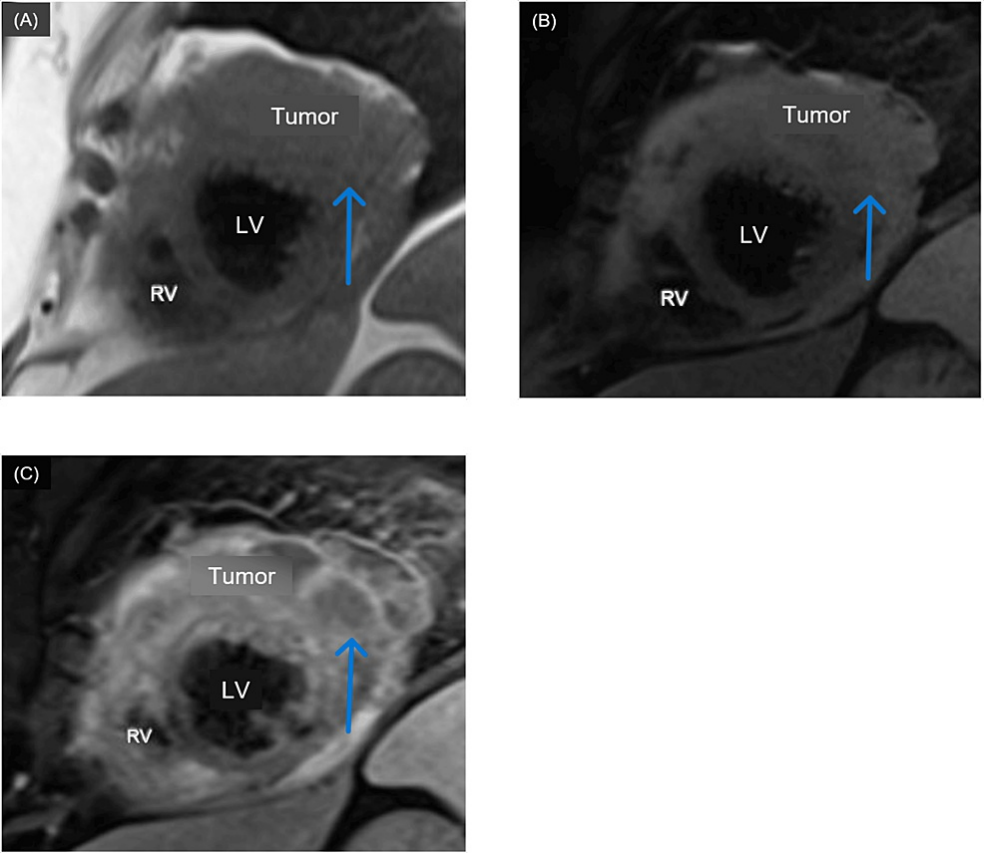
CMRI. Apical pre-gadolinium T1W slice (A), apical pre-gadolinium T2W slice (B), and apical post-gadolinium T1W slice (C). The tumor (blue arrows) is most obvious on the post-gadolinium enhancement image. LV: Left ventricle, RV: Right ventricle; CMRI: Cardiac MRI

In this case, the patient was taken for emergent coronary angiogram which demonstrated overall non-obstructive CAD. This would not have accounted for the ECG changes suggestive of significant disease relating to the lateral portion of the myocardium. CT imaging demonstrated myocardial irregularity. The CMRI findings, however, appeared to correspond to the ECG findings of lateral STEMI with tumor invasion into the anterior and lateral walls of the left ventricle (Figure 4). In general, cardiac metastasis portends a poor prognosis that is worse than with primary cardiac tumors [8]. Surgical intervention, while often purely palliative, is often undertaken to assist in mitigation of symptoms [9].

## Conclusions

This case is demonstrative of the possible relationship between ST segment elevation on ECG and corresponding tumor invasion, which was visualized on CMRI. Delineated is the need for inclusion of cardiac metastasis on the list of differential diagnoses in a patient with known history of malignancy who presents with signs of cardiac ischemia. There are a multitude of ways in which cardiac imaging should be utilized in order to arrive at the appropriate diagnosis. In a patient with non-obstructive CAD on catheterization who presents with signs and symptoms of STEMI, it may be prudent to pursue additional imaging with CT or CMRI in order to further work up and diagnose the appropriate condition, especially if cardiac metastasis is a possibility among differential diagnoses. Ultimately, however, cardiac metastasis does herald a poor prognosis and honest discussions are to be had with patients regarding palliation and goals of care.

## References

[1] Suga T, Akuzawa N, Hatori T, Imai K, Kitahara Y, Kurabayashi M (2015). ST segment elevation in secondary cardiac cancer: a case report and review of the literature. Int J Clin Exp Med.

[2] Viscuse PV, Foley TA, Michelena HI (2018). Persistent ST-segment elevation: a pandora's box. Circulation.

[3] de Bliek EC (2018). ST elevation: Differential diagnosis and caveats. A comprehensive review to help distinguish ST elevation myocardial infarction from nonischemic etiologies of ST elevation. Turk J Emerg Med.

[4] Akgun T, Gulsen K, Cinier G (2020). Electrocardiographic characteristics of metastatic cardiac tumors presenting with ST-segment elevation. J Electrocardiol.

[5] Uzun HG, Simsek E, Mammadov G, Akıllı A (2022). Inferior ST-segment elevation due to metastatic cardiac tumor. J Electrocardiol.

[6] Zhou J, Zhan C, Zhou J, Wei C, Zou C (2023). Case report: persistent ST-segment elevation due to cardiac metastasis from lung cancer. Front Cardiovasc Med.

[7] Reynen K, Köckeritz U, Strasser RH (2004). Metastases to the heart. Ann Oncol.

[8] Hoffmeier A, Schmid C, Deiters S (2005). Neoplastic heart disease-the Muenster experience with 108 patients. Thorac Cardiovasc Surg.

[9] Jung HW (2021). ST-segment elevation due to myocardial invasion of lung cancer mimicking ST elevation myocardial infarction: a case report. Medicine (Baltimore).

